# Human African trypanosomiasis: the current situation in endemic regions and the risks for non-endemic regions from imported cases

**DOI:** 10.1017/S0031182020000645

**Published:** 2020-08

**Authors:** Jiang-Mei Gao, Zheng-Yu Qian, Geoff Hide, De-Hua Lai, Zhao-Rong Lun, Zhong-Dao Wu

**Affiliations:** 1 Key Laboratory of Tropical Disease Control of the Ministry of Education, Zhongshan School of Medicine, Sun Yat-Sen University, Guangzhou 510275, China; 2 Center for Parasitic Organisms, State Key Laboratory of Biocontrol, School of Life Sciences, Sun Yat-Sen University, Guangzhou 510275, China; 3 Biomedical Research Centre and Ecosystems and Environment Research Centre, School of Science, Engineering and Environment, University of Salford, Salford, M5 4WT, UK

**Keywords:** Human African trypanosomiasis, non-endemic disease countries, sub-Saharan Africa, *T. b. rhodesiense*, *Trypanosoma brucei gambiense*

## Abstract

Human African trypanosomiasis (HAT) is caused by *Trypanosoma brucei gambiense* and *Trypanosoma brucei rhodesiense* and caused devastating epidemics during the 20th century. Due to effective control programs implemented in the last two decades, the number of reported cases has fallen to a historically low level. Although fewer than 977 cases were reported in 2018 in endemic countries, HAT is still a public health problem in endemic regions until it is completely eliminated. In addition, almost 150 confirmed HAT cases were reported in non-endemic countries in the last three decades. The majority of non-endemic HAT cases were reported in Europe, USA and South Africa, due to historical alliances, economic links or geographic proximity to disease-endemic countries. Furthermore, with the implementation of the ‘Belt and Road’ project, sporadic imported HAT cases have been reported in China as a warning sign of tropical diseases prevention. In this paper, we explore and interpret the data on HAT incidence and find no positive correlation between the number of HAT cases from endemic and non-endemic countries. This data will provide useful information for better understanding the imported cases of HAT globally in the post-elimination phase.

## Introduction

Human African trypanosomiasis (HAT), also known as sleeping sickness, is a neglected tropical disease, which is found in 36 African endemic countries (Simarro *et al*., 2010). It is caused by subspecies of the parasitic protozoan *Trypanosoma brucei* and transmitted by tsetse flies. Two subspecies are able to infect humans: *Trypanosoma brucei gambiense* causes a chronic form of HAT in West and Central Africa, while *Trypanosoma brucei rhodesiense* is the pathogenic agent for the acute form of the disease in Eastern Africa (Stich *et al*., 2002; Barrett *et al*., 2003). In the last decade of the 20th century, the number of reported cases of HAT reached alarming levels with up to 50 000 new cases reported each year (Hide, 1999). Therefore, surveillance and control activities against HAT were reinforced at the beginning of the 21st century. These effective measures enabled a steady decrease in HAT cases (WHO, 2013*a*; Franco *et al*., 2014*a*). As a consequence, HAT was included in the WHO NTD roadmap of diseases targeted for elimination by 2020. The WHO definition of elimination for HAT is that the incidence is less than one new case per 10 000 population in at least 90% of foci and with fewer than 2000 cases reported globally. According to this definition, the criteria for elimination was achieved in 2017.

It is well-known that the prevalence of the pathogen, *T. brucei* is restricted to sub-Saharan Africa, due to the distribution limitations of its transmission vector, the tsetse flies of the genus *Glossina* (Migchelsen *et al*., 2011). However, human population movements, including travelling, business connections and migration may put people with little awareness of HAT in danger. This is evidenced by reports of HAT-positivity in some travellers, military personnel and immigrants. During the period 2000–2010, more than 100 cases of HAT were reported in 19 non-disease endemic countries (DECs) (Simarro *et al*., 2012). According to the UN World Tourism Organization, the number of international tourist arrivals increased from 25 million in 1950 to 1035 million in 2012 (Norman *et al*., 2015). The strongest growth in international tourist arrivals was recorded in Asia, followed by the Pacific Region, Africa and the Americas. According to the Chinese government, the number of Chinese people living overseas has reached 60 million. Furthermore, approximately 122 million Chinese people travelled overseas in 2016. Additionally, a large number (up to 52.67 million) non-Chinese travellers crossed China's borders in 2014. Sino-overseas cooperation, triggered by the ‘Belt and Road Initiative’, has promoted the international movement of a huge population of both Chinese and non-Sino peoples and this will greatly increase the importation risk of tropical diseases that were not endemic in China.

In this article, we critically analyse the incidence of human African trypanosomiasis (HAT) in endemic countries and non-endemic countries during the period between 1990 and 2018.

## Search strategy and selection criteria

We searched PubMed for the terms ‘*Trypanosoma brucei*’, ‘*Trypanosoma brucei gambiense*’, ‘*Trypanosoma brucei rhodesiense*’, ‘Human African trypanosomiasis’ to identify papers published between Jan 1, 1990 and Dec 31, 2018 on Human African trypanosomiasis cases in non-endemic countries. Additionally, we reviewed relevant articles cited in those references and included them as primary sources when appropriate.

### Incidence of human African trypanosomiasis in endemic countries from 1990 to 2018

**Fig. 1.**
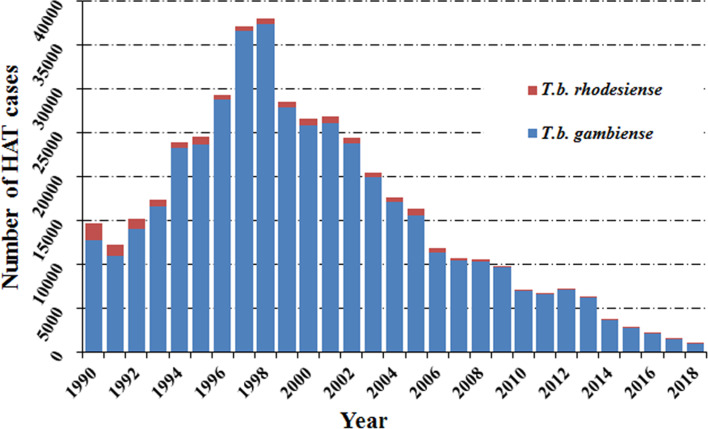
Total number of reported cases of HAT in endemic countries from 1990 to 2018.

*Note*: Data are taken from the World Health Organization.

Human African trypanosomiasis was first described around the 14th century, but it can probably be considered as endemic in African regions since the appearance of humans. In the 20th century, because of the socio-economic impact caused by HAT, effective measures were taken to control the disease. This resulted in it reaching a very low, generalized, transmission by the mid-1960s with a minimum of 4,435 cases declared in Africa in 1964 (WHO, 2013*b*). However, post-1960s, disease control interventions were constrained due to conflicts, insecurity and other social instability factors which largely resulted from the inevitable (and justifiable) emergence of independent African states from their former European colonizers. Unintentionally, this led to a resurgence of the disease in the 1980s and 1990s (Franco *et al*., 2014*a*, 2014*b*). As a consequence, the number of sleeping sickness cases reached alarming levels such that by 1998 the newly reported cases were up to 37 991 and the total number of existing cases of HAT was estimated at 300 000 cases (WHO, 1998). At that time, the status of HAT triggered attention from the health authorities in endemic countries alongside bilateral and multilateral agencies (Franco *et al*., 2014*a*, 2014*b*). The World Health Organization (WHO) also responded to this situation by coordinating international partners working in HAT control, raising awareness and political will, and bringing new resources from the public and private sectors to support national control programs. Control programs were reinforced again and as a result, in the past 20 years, important advances have been made in controlling this disease. A substantial, steady reduction in the number of cases has been observed, reaching the historical lowest figure in 2018 (Fig. 1).

**Fig. 2.**
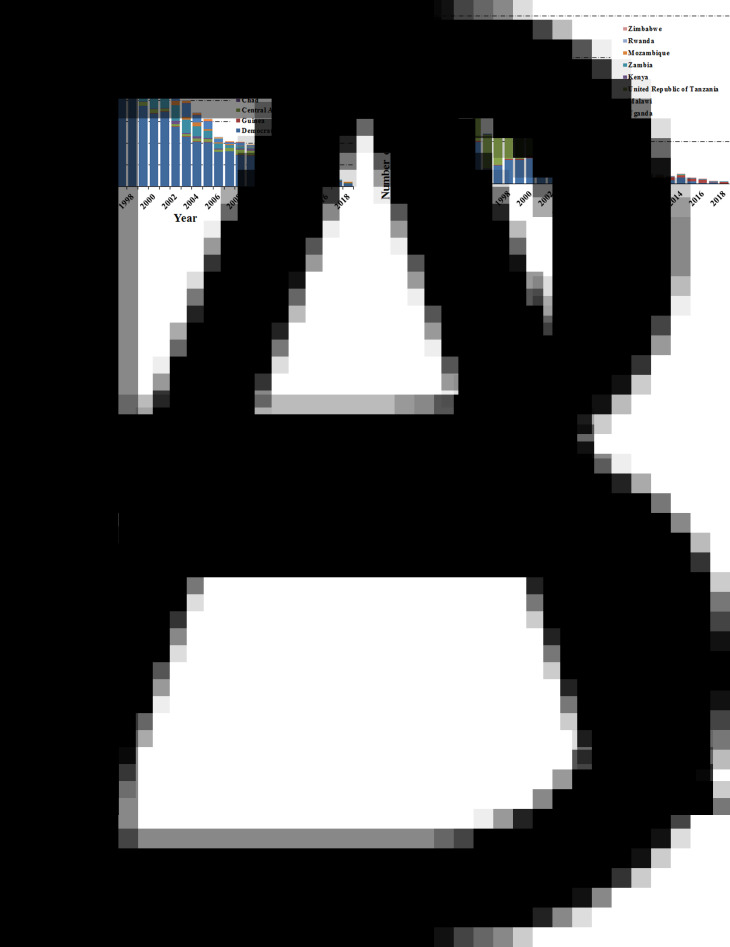
(A) Number of reported cases of *gambiense*-HAT per year from 1990 to 2018 in endemic countries with a breakdown for each country. (B) Number of reported cases of *rhodesiense*-HAT per year from 1990 to 2018 in endemic countries with a breakdown for each country. Data taken from the World Health Organization (WHO).

Among the two species which cause human African trypanosomiasis, more than 90% of HAT cases were caused by *T. b. gambiense* (Fig. 2A and B). It is worth noting that the cases in the Democratic Republic of the Congo (DRC) consistently accounted for the vast majority of *gambiense-*HAT cases. Especially in 1997 and 1998, increases in new cases of *gambiense-*HAT (25 094 and 26 318) in the DRC, led to a sharp increase in total number of *gambiense-*HAT cases. In addition, new cases of *gambiense-*HAT in Angola also sharply increased during this period due to civil war, like 6726, 8275 and 6610 new cases were reported in 1996, 1997 and 1998, respectively (Fig. 2A, Table S1). However, the total number of reported cases of rhodesiense HAT has shown a gradual descending trend over nearly three decades. We found that almost all of the new cases were reported in Uganda and United Republic of Tanzania, and the number of cases in both countries accounted for over 80% of all cases from 1990 to 2007 (Fig. 2B, Table S2). By 2008, the number of newly reported cases dropped below 200 and less than 100 new cases were found in 2011.

**Fig. 3.**
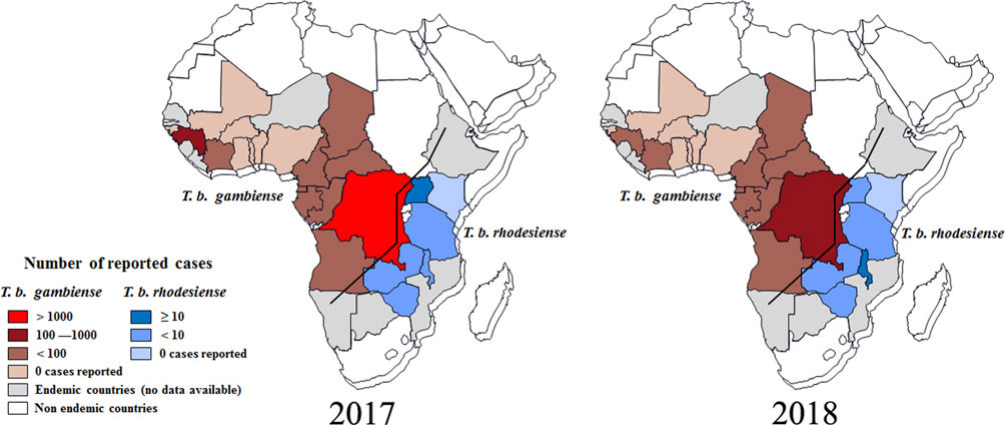
Distribution of human African trypanosomiasis in endemic countries, 2017 and 2018. Data are taken from the World Health Organization (WHO).

According to reports from WHO, less than 1500 new cases of HAT (including 1420 new cases of *T. b. gambiense* (WHO, 2019*a*), representing about 98% of the total HAT cases, and 27 new cases of *T. b. rhodesiense* (WHO, 2019*b*), representing less than 2% of the total HAT cases were recorded in 2017 (Tables S1 and S2). As always, the majority of *gambiense*-HAT cases were distributed in the Democratic Republic of the Congo with up to 1110 cases (78% of the total *gambiense*-HAT) (Fig. 3). Furthermore, 140 and 76 new cases of *gambiense*-HAT were reported in Guinea and the Central African Republic, respectively. For the rest, less than 50 new cases of *gambiense-*HAT, were reported in Angola (18 cases), Cameroon (5 cases), Chad (28 cases), Congo (15 cases), Côte d'Ivoire (3 cases), Equatorial Guinea (4 cases), Gabon (9 cases) and South Sudan (12 cases), respectively. To our knowledge, there are no reported *gambiense*-HAT cases in other West and Central Africa countries. However, during 2017, there were only 27 reported new cases of *rhodesiense*-HAT in Eastern African endemic areas, these were distributed in Uganda (13 cases), Malawi (7 cases), United Republic of Tanzania (3 cases), Zambia (3 cases) and Zimbabwe (1 case), respectively.

Based on the latest reports from WHO (WHO, 2019*a*, 2019*b*), the case numbers of HAT were down to their lowest-ever levels in 2018 and the total number of new cases was less than 1000, including 953 cases of *T. b. gambiense*, and 24 new cases of *T. b. rhodesiense*. It was exciting that the number of reported *gambiense*-HAT cases from the Democratic Republic of the Congo was down to 660 and the HAT cases from every other country numbered fewer than 100 (Fig. 3B). Surprisingly, the case number in Angola increased from 18 (2017) to 79 (2018). For *T. b. rhodesiense*, new cases were only reported in Malawi (15 cases), Uganda (4 cases) and Zambia (5 cases), respectively in 2018.

### Cases of human African trypanosomiasis reported in non-DECs

**Fig. 4.**
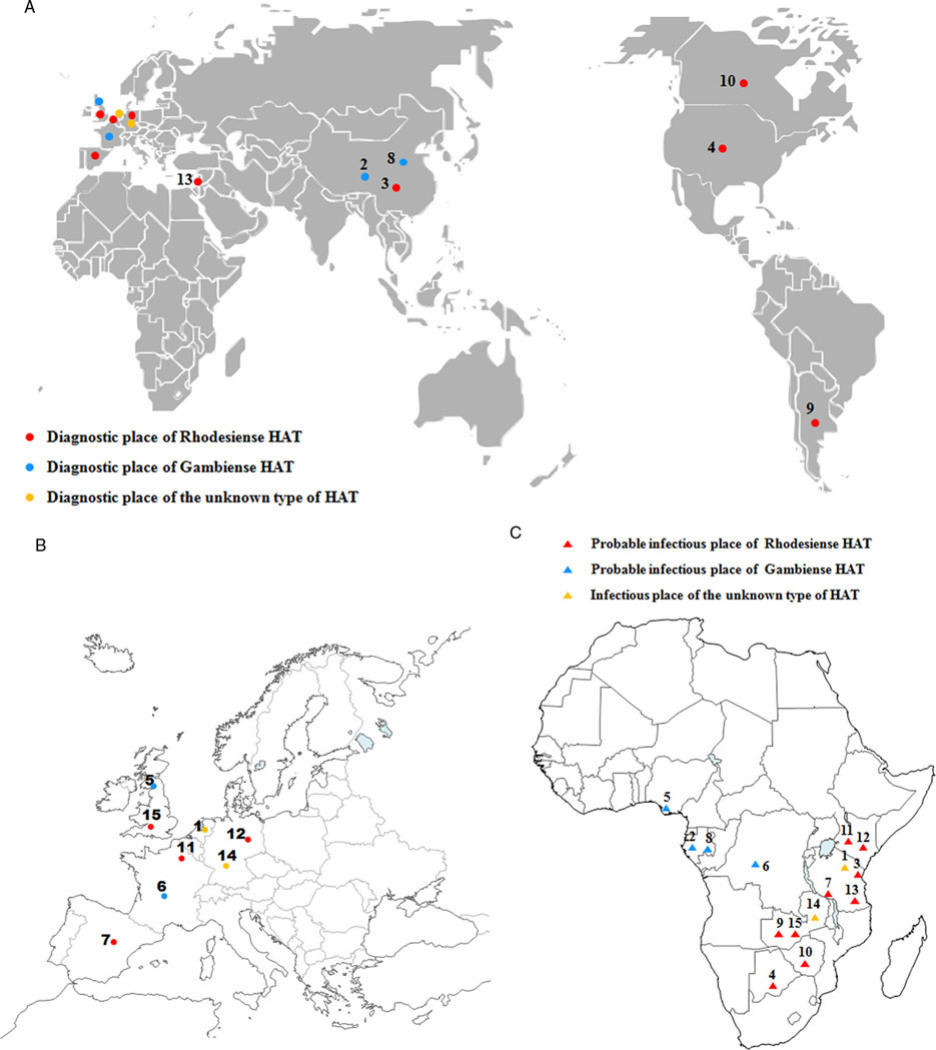
HAT cases that were diagnosed and confirmed in non-endemic countries from 2011 to 2018. (A) (B) Dots represent the place of diagnosis; (C) Triangles indicate the probable place of infection. Numbers refer to the designations in Table 1.

The human African trypanosomiasis cases in non-endemic countries from 1990 to 2010 have been collected in previous reports (Migchelsen *et al*., 2011; Simarro *et al*., 2012). Therefore, we will concentrate on cases in non-endemic countries reported between 2011 and 2018. A total of 15 cases of HAT were reported in 11 non-endemic countries (Fig. 4; Table 1), including 9 cases of *rhodesiense*-HAT and 4 cases of *gambiense*-HAT and another 2 indeterminate cases (Table 1). Rhodesiense-HAT was mainly diagnosed in tourists after short visits to endemic countries, and the majority of them were in the first stage of the disease. However, the majority of *gambiense*-HAT patients had been living in endemic countries for an extended period for various reasons (e.g. migrants or expatriates, etc). Therefore, by the time they were diagnosed, the disease had developed into the second stage. Due to historical or economic links with endemic countries and frequent travel to endemic countries, citizens from Europe have more chances of becoming infected with trypanosomes. More than half of the cases (8 cases, 53.3% of total) were diagnosed in European countries (including Belgium, 1 case; France, 1 case; Germany, 2 cases; Netherlands, 1 case; Spain, 1 case; and UK, 2 cases). In addition, another 2 cases were reported in North America (including one in Canada and one in USA) and South America (Argentina), respectively. The remaining 4 cases were identified in Asia. Interestingly, with the exception of 1 case from Israel, the other 3 HAT cases were all diagnosed in China, which is now starting to appear as a non-DEC with increasing numbers of imported HAT cases during the period of 2011–2018.

**Table 1.** Cases of human African trypanosomiasis (HAT) recorded in non-endemic countries from 2011 to 2018

Cases^Refs^	Time		Place of diagnosis	Place of infection	Sex/age	Activity	Stage	Chancre	Species
1^(Wijsman *et al*., 2018)^	2018^a^	NA	Netherlands	Tanzania and Kenya	W56	Traveller	1	Yes	*T. b.* (unknown)
2^(Wang *et al*., 2018)^	2017	August	China	Libreville，Gabon	M60	worker	2	NA	*T. b. gambiense*
3^(Liu *et al*., 2018)^	2017	August	China	Masai Mara area in Kenya and the Serengeti area in Tanzania	W41	Traveller	1	Yes	*T. b. rhodesiense*
4^(Streit and Matsumoto, 2016)^	2016^a^	December	USA	Botswana and Zimbabwe	W60	Traveller	1	NA	*T. b. rhodesiense*
5^(Luintel *et al*., 2017)^	2016	August	UK	Delta State, Nigeria	W58	NA	2	NA	*T. b. gambiense*
6^(De Kyvon *et al*., 2016)^	2016^a^	May	France	Democratic Republic of the Congo	M14month	Delivery	2	NA	*T. b. gambiense*
7^(Gomez-Junyent *et al*., 2017)^	2015	August	Spain	Tanzania	W49	Traveller	1	Yes	*T. b. rhodesiense*
8^(Hu *et al*., 2016)^	2014	September	China	Gabon	M45	Sailor	2	Yes	*T. b. gambiense*
9^(Pale and Vigna, 2013)^	2013^a^	August	Argentina	Zambia	M65	Traveller	NA	NA	*T. b. rhodesiense*
10^(Pasternak *et al*., 2013)^	2013^a^	January	Canadian	Zimbabwe	M	Traveller	1	NA	*T. b. rhodesiense*
11^(Clerinx *et al*., 2012)^	2012	February	Belgium	Masai Mara National Reserve in Kenya	M	Traveller	NA	Yes	*T. b. rhodesiense*
12^(Wolf *et al*., 2012)^	2012	January	Germany	Masai Mara area in Kenya	M62	Traveller	1	NA	*T. b. rhodesiense*
13^(Meltzer *et al*., 2012)^	2012^a^	NA	Israeli	Tanzania	W31	Traveller	1	NA	*T. b. rhodesiense*
14^(Richter *et al*., 2012)^	2012^a^	March	Germany	Zambia	M58	Traveller	NA	NA	*T. b.* (unknown)
15^(Cottle *et al*., 2012)^	2012^a^	February	UK	Zambia	W49	Traveller	1	NA	*T. b. rhodesiense*

NA: Information is unavailable on the case report.

a Time refers to the published date when the diagnostic date is unavailable.

**Fig. 5.**
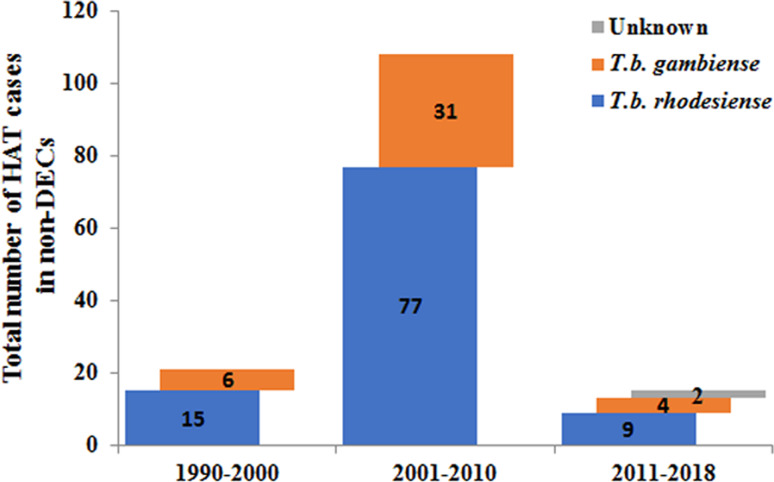
Number of HAT cases diagnosed in non-endemic countries in different periods.

**Fig. 6.**
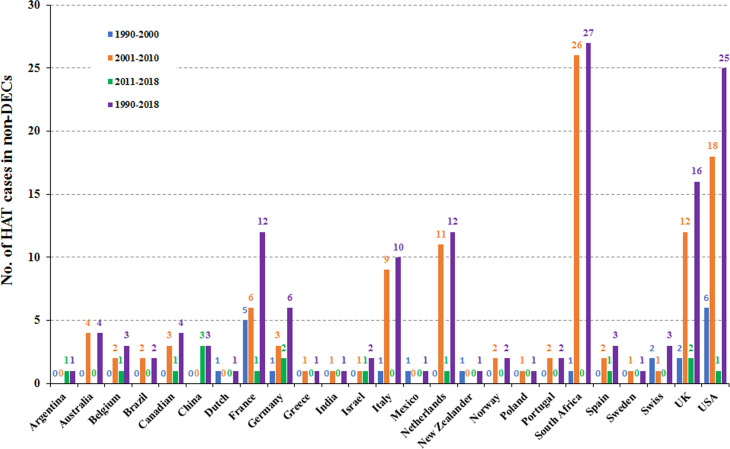
HAT cases reported in non-endemic countries in different time periods.

Furthermore, the number of reported cases diagnosed in non-endemic countries between 2011 and 2018 were compared with previous records (Fig. 5). It is reported that many more HAT cases were diagnosed in non-endemic countries from 2001 to 2010 including 77 cases of *rhodesiense*-HAT and 31 cases of *gambiense*-HAT, as detailed in supplementary Table S3. As shown in Fig. 6, during this period of time, many imported HAT cases were diagnosed in South Africa (26 cases, accounting for 24.1% of the total 108 cases). This is probably due to its proximity to endemic countries with famous protected areas and game reserves. In addition, due to historical or economic links with endemic countries, most of the HAT cases were diagnosed in North American and European countries, 21 and 53 cases, account for 19.4% and 49.1%, respectively. Among them, the USA and UK contributed significant numbers of imported HAT cases (USA, 18 cases; and the UK, 12 cases), followed by Netherlands (11), Italy (9), France (6) and Germany (5).

Only 21 cases of HAT (15 cases of *rhodesiense*-HAT and 6 cases of *gambiense*- HAT) were reported in non-endemic countries between 1990 and 2000 (Fig. 5, Table S4). Among them, most cases were mainly diagnosed in the USA (6) and France (5), with the rest being diagnosed in the Netherlands (1), Germany (1), Italy (1), Mexico (1), New Zealand (1), South Africa (1), Switzerland (2) and the UK (2) (Fig. 6). There was a resurgence of HAT in the 1990s when the number of sleeping sickness cases reached the highest ever recorded in 1998. Why there is no positive correlation between the number of HAT cases in non-endemic countries and endemic countries is puzzling? It may be that some HAT cases were not published in the literature or perhaps there have been some misdiagnosed cases in the past. However, from the beginning of this century, pharmacy services in non-endemic countries have had to request drugs from WHO to treat HAT cases and, at the same time, provide epidemiological, parasitological, biological, and clinical data. Therefore, this information has enabled WHO to maintain HAT surveillance system and a comprehensive database for non-endemic countries.

### The sources of imported HAT cases in non-endemic regions

During the period from 1990 to 2018, more than 101 reported *rhodesiense*-HAT cases were exported from eastern and southern Africa (As shown in Table 2). It is supposed that the infection has been contracted in protected areas such as national parks (NP), wildlife reserves, and game reserves (Simarro *et al*., 2012; Selby *et al*., 2013). The country exporting the majority of rhodesiense HAT cases is the United Republic of Tanzania, with up to 56.4% (57 cases). Other exporting countries are Malawi (15%, 15 cases), Zambia (9.9%, 10 cases), Zimbabwe (6%, 6 cases), Rwanda (5%, 5 cases), Kenya (3%, 3 cases) and Uganda (2%, 2 cases). However, the number of rhodesiense HAT endemic cases in a country does not positively correlate with the number of cases exported. For example, from 1990 to 2000, there were no new cases reported in Rwanda, nevertheless, it exported more than 5 cases to non-endemic areas from this country; while the maximum number of endemic cases was reported in Uganda, but no cases were exported from this country. In addition, only 8 cases were reported in Zimbabwe from 2001 to 2010, but as many as 5 cases were exported during this period. Furthermore, the number of exported cases from Kenya is the same as the number of domestic cases from 2011 to 2018. Perhaps the factors underlying the export of cases are related more to the awareness of travellers and the messaging around risk in specific countries than biological factors such as the epidemiology of exposure.

**Table 2.** Summary of *rhodesiense*-HAT cases in eastern African countries but reported in endemic countries or non-endemic countries

	1990–2000		2001–2010		2011–2018		Total in non-DECs
	DECs	Non-DECs	DECs	Non-DECs	DECs	Non-DECs	
Kenya	164	1	23	0	2	2	3
Malawi	736	1	465	14	180	0	15
Rwanda	0	5	0	0	0	0	5
Tanzania	3424	6	1294	49	16	2	57
Tanzania or Kenya	••	••	••	••	••	1	1
Tanzania, Kenya or Rwanda	••	1	••	••	••	••	1
Uganda	5458	0	2660	2	319	0	2
Zambia	41	1	81	7	42	2	10
Zimbabwe	10	0	8	5	22	1	6
Zimbabwe or Botswana	••	••	••	••	••	1	1
Total in non-DECs	••	15	••	77	••	9	101

DECs, disease endemic countries; Non-DECs, non-disease endemic countries; ••, No data.

However, the majority of HAT cases were *gambiense*-HAT that were reported in western and central Africa, whereas there were only 40 cases of exported *gambiense*-HAT during 1990–2018 (Table 3). The countries of origin for exported *gambiense*-HAT are mainly the Gabon, Angola and DRC, each accounting for 25% (10 cases), 20% (8 cases) and 20% (8 cases), followed by Cameroon (10%, 4 cases), Equatorial Guinea, Nigeria, Central African Republic and Uganda (5%, 2 cases each), Sudan (2.5%, 1 case) and one uncertain case (2.5%) which may have originated from Nigeria or Gabon. To our surprise, as many as 7 and 3 cases were exported from Gabon and Cameroon, respectively, despite numbers of indigenous cases being as low as 305 and 174 in these two countries from 2001 to 2010. In addition, although only 6 indigenous cases were reported in Nigeria, 1 case of exported HAT has been reported between 2011 and 2018. There seems to be a general lack of association between incidence and exported infections across many endemic countries.

**Table 3.** Summary of *gambiense*-HAT cases in west and central African countries but reported in endemic countries or non-endemic countries

	1990–2000		2001–2010		2011–2018		Total in non-DECs
	DECs	Non-DECs	DECs	Non-DECs	DECs	Non-DECs	
Angola	43 017	1	18 048	7	402	0	8
Cameroon	360	1	174	3	52	0	4
Central African Republic	6320	0	6990	2	1090	0	2
DRC	176 073	1	99 451	6	25 635	1	8
Eq. Guinea	515	0	165	2	13	0	2
Gabon	451	1	305	7	81	2	10
Nigeria	65	1	107	0	6	1	2
Nigeria or Gabon	••	1	••	••	••	••	1
Sudan	6073	0	14 149	1	843	0	1
Uganda	14 790	0	2928	2	90	0	2
Total in non-DECs	••	6	••	30	••	4	40

DECs, disease endemic countries; Non-DECs, non-disease-endemic countries; ••, No data.

## HAT carriers may not be the main infective source for non-endemic countries

**Fig. 7.**
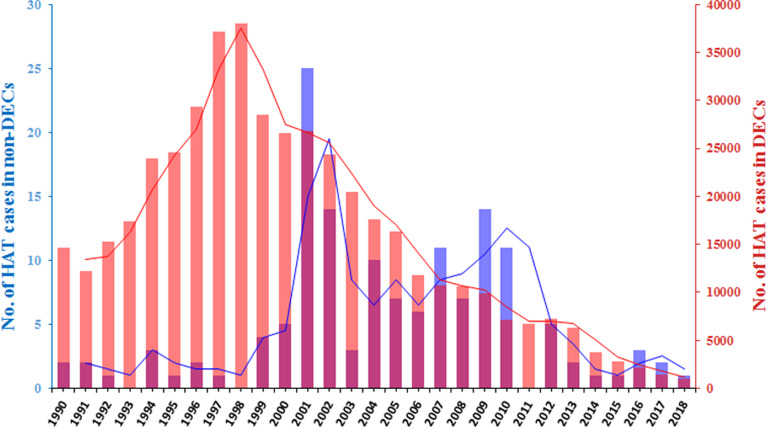
The dynamic trend changes in HAT cases diagnosed in non-DECs and DECs. Bar graphs indicate the annual figures. The line graphs indicate the distribution of 3-year averages.

In an analysis of the dynamic change trend of HAT cases diagnosed in non-endemic countries and endemic countries, we found that there is no positive correlation (*P* = 0.62) between the two groups (Fig. 7). It is possible that the number of people travelling to eastern Africa, endemic areas of the *rhodesiense*-HAT, is higher than that in other regions. This suggestion is supported by the most recent tourism data (2018) that shows higher numbers of incoming tourists in East African DEC countries (e.g. Malawi, 870k; Zambia, 1072k; Tanzania, 1506k) compared with West African DEC countries [e.g. DRC, 351k (2016 data); Congo 158k] (UNWTO, 2020). However, more than 70% of the cases reported from non-endemic countries were infected during trips to east African countries, in particular to popular tourist countries, such as Tanzania, Malawi or Zambia. By comparison, the overall proportion of *rhodesiense*-HAT cases reported from endemic countries accounts for only 10% of the total number of cases.

Interestingly, an inspection of the data presented in Fig. 7 shows that peaks of reported non-DEC infections occur a few years after the corresponding peaks in DECs (see for example the endemic disease peak in 1997–1998 with the peak of reported cases in non-DECs 2001–2002, 4 years later). This suggests that there might be a time delay between the local disease status in endemic countries and the recording of reported cases in non-DECs. Analysis of correlation between the number of cases in DECs with those reported from non-DECs shows a highly significant correlation (*P* = 0.0011) when the comparative datasets are offset by 4 years (i.e. DEC data from 1990 to 2014 compared year by year with non-DEC data from 1994 to 2018). Significant correlations are additionally found when comparisons are offset by 3–7 years. Such a delay between the endemic peaks could be attributed to the relatively long incubation period in *T. b. gambiense* infections, a delay in diagnosis or reporting of cases in the recipient non-DEC or a generic delay in reporting back to WHO. Comparable significant correlations were found when this analysis was carried out separately on *T. b. gambiense* data (e.g. *P* = 0.0009; 4 year offset data) but no correlation at all was seen when analysing *T. b rhodesiense* non-DEC case data (e.g. *P* = 0.83; 4 year offset data). This broad analytical approach has many flaws but suggests that more detailed case information and better stratification of the data might tell us more about the role of HAT carriers or the reporting processes in non-DEC cases.

In addition to human reservoirs, many livestock and wildlife species can act as animal reservoirs, from which *T. b. gambiense* and *T. b. rhodesiense* maybe spill over to humans (Yesufu, 1971; Molyneux, 1973; Mehlitz, 1977; Kageruka, 1989; Jamonneau *et al*., 2004; Njiokou *et al*., 2006; Simarro *et al*., 2010; Cecchi *et al*., 2014, 2015; Cordon-Obras *et al*., 2015; Büscher *et al*., 2017; N'Djetchi *et al*., 2017). It has been demonstrated that human-derived *T. b. gambiense* strains that were cyclically transmitted by tsetse flies between animals for more than a year remained transmissible to humans (Van Hoof, 1947). Compared to latent infections in humans, our current knowledge of *T. b. gambiense* and *T. b. rhodesiense* infections in animals are very limited and fragmented. Therefore, it is not known whether cryptic reservoirs in animals will compromise the sustainable elimination of human Africa trypanosomiasis.

### The risk of mechanical transmission of Trypanosoma spp.

It is known that tsetse flies play the most important role, as cyclical vectors, in the transmission of African human and livestock trypanosomes (*Trypanosoma congolense*, *T. vivax*, and *T. brucei*) (Hoare, 1972; Desquesnes and Dia, 2003*a*, 2003*b*). However, previous research has demonstrated that many species of *Trypanosoma* can be mechanically transmitted by haematophagous insects or sucking flies (Desquesnes and Dia, 2003*a*, 2003*b*). Importantly, *T. brucei rhodesiense* was reported to be mechanically transmitted by *Musca sorbens*, which could potentially pose a threat to human populations and livestock in non-endemic areas (Simarro *et al*., 2010; Büscher *et al*., 2018).

### China as a new destination for imported cases

Until recently, there have been no reported cases of trypanosomiasis in China. As China further develops international connections through economic development and tourism in African countries, the risk of the emergence of non-endemic cases in China will increase. Such a development may require greater awareness, in the Chinese health systems, of the possible emergence of cases. Strikingly, there have been three cases of imported human African trypanosomasis in recent years, including 2 cases of *T. b. gambiense*, discovered in Chinese workers and presumably infected in Gabon, Western Africa; whereas another case of *T. b. rhodesiense* was found in a Chinese tourist visiting game parks in Tanzania, Eastern Africa (Zhou *et al*., 2018). According to the Chinese government, approximately 60 million Chinese people are now living overseas and in 2016, more than 122 million Chinese people travelled overseas (IOMMA, 2017). Additionally, the number of visitors entering China numbered up to 52.67 million in 2014 (CNTA, 2017). Therefore, economic globalization and population migration will increase the importation risk of tropical diseases that were not previously endemic in China and, of course, other countries too. This situation may be mitigated as African countries are now moving into the elimination phase of sleeping sickness but the situation needs to be monitored in the possible event of any post-elimination resurgence of the disease.

### Concluding remarks

Sleeping sickness, also known as HAT, is a neglected disease that impacts 70 million people living in 1.55 million km^2^ in sub-Saharan Africa (Aksoy *et al*., 2017). The successful control activities carried out over the last less 20 years have substantially reduced the burden of disease. According to the latest report from WHO (WHO, 2019*a*, 2019*b*), new cases of HAT were less than 1000 in 2018 (98% of total HAT is *T. b. gambiense*, while *T. b. rhodesiense* is less than 2%). However, in some rural areas where trypanosomiasis is prevalent, the overall performance of peripheral health systems is often weak and characterized by unskilled staff, low attendance and low coverage.

Furthermore, political instability and insecurity are inescapable challenges in an elimination programme. Gambiense-HAT does not spread easily and humans are the only significant reservoir. The successful elimination of *gambiense*-HAT requires a turmoil-free socio-political context. However, the elimination of *rhodesiense*-HAT poses challenges because of the vast animal reservoirs.

Therefore, the strengthening of health systems to implement the activities included in the elimination strategies is essential. Past and current experiences show that HAT elimination is difficult and will require more effort, time, and money than initially anticipated. History teaches us that falling case numbers can result in a decline in donor and control agency interest, opening the door to swift and severe recrudescence (Büscher *et al*., 2017). Advocacy is needed to sensitize donors on the importance of switching from control to an elimination mindset.

It is known that the majority of HAT cases reported by endemic countries correspond to the gambiense form (98%), the opposite is true for imported cases in non-endemic countries, where 72% of cases are caused by *T. b. rhodesiense* and 28% by *T. b. gambiense*. However, the proportion of gambiense- to rhodesiense-HAT cases diagnosed in non-endemic countries is lower than one would probably expect. Moreover, it is difficult to establish the number of migrants and refugees travelling to non-endemic countries from HAT transmission areas, and even more difficult to ascertain how many of them are affected by HAT. Therefore, it is a potential threat to non-endemic countries because global mobility means that *Trypanosoma brucei* can be spread in many ways.

## Supplementary material

For supplementary material accompanying this paper visit https://doi.org/10.1017/S0031182020000645. click here to view supplementary material
