# Supplementary material for: Human African trypanosomiasis: the current situation in endemic regions and the risks for non-endemic regions from imported cases
Source: Parasitology. 2020 Apr 27;147(9):922–31. doi: 10.1017/S0031182020000645 (PMC7391876; doi:10.1017/S0031182020000645)
Supplement: Supplementary file 1 [file S0031182020000645sup001.docx]

**Table S1. New cases of *gambiense-*human African trypanosomiasis in Disease Endemic Countries (DECs) reported from 1990 to 2018.**

|  | **1990** | **1991** | **1992** | **1993** | **1994** | **1995** | **1996** | **1997** | **1998** | **1999** | **2000** | **2001** | **2002** | **2003** | **2004** | **2005** | **2006** | **2007** | **2008** | **2009** | **2010** | **2011** | **2012** | **2013** | **2014** | **2015** | **2016** | **2017** | **2018** |
| --- | --- | --- | --- | --- | --- | --- | --- | --- | --- | --- | --- | --- | --- | --- | --- | --- | --- | --- | --- | --- | --- | --- | --- | --- | --- | --- | --- | --- | --- |
| Angola | 1498 | 2094 | 2406 | 1796 | 1274 | 2441 | 6726 | 8275 | 6610 | 5351 | 4546 | 4577 | 3621 | 3115 | 2280 | 1727 | 1105 | 648 | 517 | 247 | 211 | 154 | 70 | 69 | 36 | 35 | 20 | 18 | 79 |
| Benin | 0 | 0 | 2 | 1 | 0 | 0 | 0 | 0 | 0 | 20 | 0 | 0 | 0 | 0 | 0 | 0 | 0 | 0 | 0 | 0 | 0 | 0 | 0 | 0 | 0 | 0 | 0 | 0 | 0 |
| Burkina Faso | 27 | 27 | 20 | 17 | 18 | 13 | 12 | 1 | 15 | 15 | 0 | 0 | 0 | 0 | 0 | 0 | 0 | 0 | 0 | 0 | 0 | 0 | 0 | 0 | 0 | 1 | 0 | 0 | 0 |
| Cameroon | 86 | 69 | 21 | 3 | 20 | 21 | 17 | 10 | 54 | 32 | 27 | 14 | 32 | 33 | 17 | 3 | 15 | 7 | 13 | 24 | 16 | 15 | 7 | 6 | 7 | 6 | 6 | 5 | 7 |
| Central African Republic | 308 | 197 | 362 | 262 | 368 | 676 | 492 | 730 | 1068 | 869 | 988 | 718 | 572 | 539 | 738 | 666 | 460 | 654 | 1194 | 1054 | 395 | 132 | 381 | 59 | 194 | 147 | 101 | 76 | 57 |
| Chad | 20 | 221 | 149 | 65 | 214 | 315 | 178 | 122 | 134 | 187 | 153 | 138 | 715 | 222 | 483 | 190 | 276 | 97 | 196 | 510 | 232 | 276 | 197 | 195 | 95 | 67 | 54 | 28 | 12 |
| Congo | 580 | 703 | 727 | 829 | 418 | 475 | 474 | 142 | 201 | 91 | 111 | 894 | 1005 | 717 | 873 | 398 | 300 | 189 | 182 | 87 | 87 | 61 | 39 | 20 | 21 | 36 | 18 | 15 | 24 |
| Côte d'Ivoire | 365 | 349 | 456 | 260 | 206 | 326 | 240 | 185 | 121 | 104 | 188 | 92 | 97 | 68 | 74 | 42 | 29 | 13 | 14 | 8 | 8 | 10 | 9 | 7 | 6 | 3 | 0 | 3 | 2 |
| Democratic Republic of the Congo | 7515 | 5825 | 7757 | 11384 | 19021 | 18182 | 19342 | 25094 | 26318 | 18684 | 16951 | 17300 | 13816 | 11459 | 10339 | 10249 | 8013 | 8155 | 7318 | 7178 | 5624 | 5590 | 5968 | 5647 | 3205 | 2347 | 1768 | 1110 | 660 |
| Equatorial Guinea | 63 | 36 | 45 | 30 | 85 | 37 | 46 | 67 | 62 | 28 | 16 | 17 | 32 | 23 | 22 | 17 | 13 | 15 | 11 | 7 | 8 | 1 | 2 | 3 | 0 | 0 | 3 | 4 | 4 |
| Gabon | 80 | 45 | 33 | 80 | 61 | 20 | 32 | 11 | 6 | 38 | 45 | 30 | 26 | 26 | 49 | 53 | 31 | 30 | 24 | 14 | 22 | 17 | 9 | 17 | 10 | 9 | 10 | 9 | 16 |
| Gambia | - | - | - | - | - | - | - | - | - | - | - | - | - | - | - | - | - | - | - | - | - | - | - | - | - | - | - | - | - |
| Ghana | 3 | 6 | 16 | 0 | 0 | 0 | 1 | 0 | 0 | 0 | 1 | 0 | 0 | 0 | 0 | 0 | 0 | 0 | 0 | 0 | 0 | 0 | 0 | 1 | 0 | 0 | 0 | 0 | 0 |
| Guinea | 52 | 29 | 24 | 27 | 26 | 33 | 38 | 88 | 99 | 68 | 52 | 72 | 132 | 130 | 95 | 94 | 48 | 69 | 90 | 79 | 68 | 57 | 70 | 78 | 33 | 29 | 108 | 140 | 74 |
| Guinea-Bissau | - | - | - | - | - | - | - | - | - | - | - | - | - | - | - | - | - | - | - | - | - | - | - | - | - | - | - | 0 | - |
| Liberia | - | - | - | - | - | - | - | - | - | - | - | - | - | - | - | - | - | - | - | - | - | - | - | - | - | - | - | - | - |
| Mali | 0 | 0 | 0 | 27 | 17 | 11 | 0 | 0 | 0 | 0 | 0 | 0 | 0 | 0 | 0 | 0 | 0 | 0 | 0 | 0 | 0 | 0 | 0 | 0 | 0 | 0 | 0 | 0 | 0 |
| Niger | - | - | - | - | - | - | - | - | - | - | - | - | - | - | - | - | - | - | - | - | - | - | - | - | - | 0 | - | - | - |
| Nigeria | 24 | 0 | 0 | 0 | 0 | 0 | 0 | 0 | 0 | 27 | 14 | 14 | 26 | 31 | 10 | 21 | 3 | 0 | 0 | 0 | 2 | 3 | 2 | 0 | 0 | 0 | 1 | 0 | 0 |
| Senegal | - | - | - | - | - | - | - | - | - | - | - | - | - | - | - | - | - | - | - | - | - | - | - | - | - | - | - | - | - |
| Sierra Leone | - | - | - | - | - | - | - | - | - | - | - | - | - | - | - | - | - | - | - | - | 0 | - | - | - | - | - | - | - | - |
| South Sudan | 67 | 58 | 28 | 62 | 69 | 56 | 157 | 737 | 1726 | 1312 | 1801 | 1919 | 3121 | 3061 | 1742 | 1853 | 789 | 469 | 623 | 373 | 199 | 272 | 317 | 117 | 63 | 45 | 17 | 12 | 17 |
| Togo | 2 | 0 | 0 | 0 | 0 | 3 | 0 | 0 | 0 | 0 | 0 | 0 | 0 | 0 | 0 | 0 | 0 | 0 | 0 | 0 | 0 | 0 | 0 | 0 | 0 | 0 | 0 | 0 | 0 |
| Uganda | 2066 | 1328 | 2042 | 1764 | 1469 | 1062 | 981 | 1123 | 971 | 1036 | 948 | 310 | 604 | 517 | 378 | 311 | 290 | 120 | 198 | 99 | 101 | 44 | 20 | 9 | 9 | 4 | 4 | 0 | 1 |
| Total reported | 12756 | 10987 | 14088 | 16607 | 23266 | 23671 | 28736 | 36585 | 37385 | 27862 | 25841 | 26095 | 23799 | 19941 | 17100 | 15624 | 11372 | 10466 | 10380 | 9680 | 6973 | 6632 | 7091 | 6228 | 3679 | 2729 | 2110 | 1420 | 953 |

**Table S2 New cases of *rhodesiense-*human African trypanosomiasis in DECs reported between 1990 and 2018.**

|  | **1990** | **1991** | **1992** | **1993** | **1994** | **1995** | **1996** | **1997** | **1998** | **1999** | **2000** | **2001** | **2002** | **2003** | **2004** | **2005** | **2006** | **2007** | **2008** | **2009** | **2010** | **2011** | **2012** | **2013** | **2014** | **2015** | **2016** | **2017** | **2018** |
| --- | --- | --- | --- | --- | --- | --- | --- | --- | --- | --- | --- | --- | --- | --- | --- | --- | --- | --- | --- | --- | --- | --- | --- | --- | --- | --- | --- | --- | --- |
| Botswana | - | - | - | - | - | - | - | - | - | - | - | - | - | - | - | - | - | - | - | - | - | - | - | - | - | - | - | - | - |
| Burundi | - | - | - | - | - | - | - | - | - | - | - | - | - | - | - | - | - | - | - | - | - | - | - | - | - | - | - | - | - |
| Eswatini | - | - | - | - | - | - | - | - | - | - | - | - | - | - | - | - | - | - | - | - | - | - | - | - | - | - | - | - | - |
| Ethiopia | - | - | - | - | - | - | - | - | - | - | - | - | - | - | - | - | - | - | - | - | - | - | - | - | - | - | - | - | - |
| Kenya | 91 | 8 | 4 | 2 | 1 | 0 | 2 | 5 | 14 | 22 | 15 | 10 | 11 | 0 | 0 | 0 | 1 | 0 | 0 | 1 | 0 | 0 | 2 | 0 | 0 | 0 | 0 | 0 | 0 |
| Malawi | 228 | 195 | 143 | 53 | 31 | 15 | 8 | 7 | 10 | 11 | 35 | 38 | 43 | 70 | 48 | 41 | 58 | 50 | 49 | 39 | 29 | 23 | 18 | 35 | 32 | 30 | 35 | 7 | 15 |
| Mozambique | 3 | 7 | 24 | 10 | 16 | - | - | - | - | - | - | - | 1 | - | 1 | - | - | - | - | - | - | - | - | - | - | - | - | - | - |
| Namibia | - | - | - | - | - | - | - | - | - | - | - | - | - | - | - | - | - | - | - | - | - | - | - | - | - | - | - | - | - |
| Rwanda | - | - | - | - | - | - | - | - | - | - | - | - | - | - | - | - | - | - | - | - | - | - | - | - | - | - | 0 | 0 | 0 |
| Uganda | 1417 | 832 | 606 | 503 | 342 | 497 | 178 | 217 | 283 | 283 | 300 | 426 | 329 | 338 | 335 | 473 | 261 | 119 | 138 | 129 | 112 | 84 | 71 | 43 | 70 | 28 | 10 | 13 | 4 |
| United Republic of Tanzania | 187 | 177 | 366 | 262 | 319 | 422 | 400 | 354 | 299 | 288 | 350 | 277 | 228 | 113 | 159 | 186 | 127 | 126 | 59 | 14 | 5 | 1 | 4 | 1 | 1 | 2 | 4 | 3 | 0 |
| Zambia | 7 | - | 4 | 1 | 1 | 1 | 3 | - | - | 15 | 9 | 4 | 5 | 15 | 9 | 7 | 6 | 10 | 13 | 4 | 8 | 3 | 6 | 6 | 12 | 8 | 4 | 3 | 5 |
| Zimbabwe | - | - | - | - | 1 | - | - | 9 | - | - | - | - | - | - | - | 3 | - | - | 0 | 3 | 2 | 4 | 9 | 1 | 3 | 3 | 1 | 1 | 0 |
| Total reported | 1933 | 1219 | 1147 | 831 | 711 | 935 | 591 | 592 | 606 | 619 | 709 | 755 | 617 | 536 | 552 | 710 | 453 | 305 | 259 | 190 | 156 | 115 | 110 | 86 | 118 | 71 | 54 | 27 | 24 |

**Table S3. Cases of human African trypanosomiasis (HAT) in non-endemic countries from 2001 to 2010.**

| **Year** | **Place of diagnosis** | **Place of infection** | **Sex/age** | **Activity** | **Diagnosis** | **Stage** | **Treatment** | **Species** | **Reference** |
| --- | --- | --- | --- | --- | --- | --- | --- | --- | --- |
| 2001 | Toronto Canada | DRC | M 42 | Refugee | CSF, blood, and lymph | Second | Eﬂornithine | *T. b. gambiense* | 1 |
| 2001 | New York USA | Angola | M 30 | UN staff | CSF | Second | Eﬂornithine | *T. b. gambiense* | 2 |
| 2001 | Atlanta USA | Kajo-Keji Sudan | M 19 | Refugee | CSF | Second | Eﬂornithine | *T. b. gambiense* | 3 |
| 2001 | Poitiers France | Kinshasa DRC | M 28 | Expat business | Lymph node/BM | Second | Eﬂornithine | *T. b. gambiense* | 4 |
| 2001 | Brazil | Angola | M NA | Soldier | NA | NA | NA | *T. b. gambiense* | 2 |
| 2001 | Hamburg Germany | Campo Cameroon | M 38 | Migrant | Lymph node/BM | First | Suramin | *T. b. gambiense* | 5 |
| 2002 | Canada | Zaire Angola | M 42 | Migrant | Blood and CSF | Second | Eﬂornithine | *T. b. gambiense* | 1 |
| 2002 | Paris France | Gabon | M NA | Expat forest | Blood smear | First | Pentamidine | *T. b. gambiense* | 6 |
| 2004 | Udine Italy | Komo, Gabon | M 44 | Expat forest | CSF | Second | Eﬂornithine | *T. b. gambiense* | 7 |
| 2004 | Verona Italy | CAR | F 54 | Expat missionary | Blood CCT and blood smear | First | Pentamidine | *T. b. gambiense* | 7 |
| 2004 | Netherlands | Zaire Angola | F 27 | Migrant | CSF | Second | Eﬂornithine | *T. b. gambiense* | 8 |
| 2005 | Italy | Gabon | M 44 | NA | Blood and CSF | Second | Eﬂornithine | *T. b. gambiense* | 9 |
| 2005 | Italy | CAR | F 54 | NA | Blood | First | Pentamidine, Eﬂornithine | *T. b. gambiense* | 7 |
| 2006 | Torino Italy | DRC | M 29 | Migrant | CSF | Second | Eﬂornithine | *T. b. gambiense* | 10 |
| 2007 | Bordeaux France | Gabon | M 37 | Expat forest | Lymph node | First | Pentamidine | *T. b. gambiense* | 11 |
| 2007 | Bordeaux France | Gabon | M 72 | Expat forest | Blood smear | First | Pentamidine | *T. b. gambiense* | 11 |
| 2007 | Berlin Germany | Manfe Cameroon | M NA | Migrant | CSF | Second | Eﬂornithine | *T. b. gambiense* | 10 |
| 2007 | Athens Greece | West Africa | M 65 | Sailor | Blood smear | Second | Eﬂornithine | *T. b. gambiense* | 10 |
| 2007 | Madrid Spain | Mbini Eq. Guinea | M 55 | Migrant | CSF (PCR) + serology | Second | Eﬂornithine | *T. b. gambiense* | 10 |
| 2008 | Perth Australia | Adjumani Uganda | F 19 | Refugee | CSF | Second | Eﬂornithine | *T. b. gambiense* | 12 |
| 2008 | Toronto Canada | DRC | M 20 | Refugee | Blood smear | Second | Eﬂornithine | *T. b. gambiense* | 10 |
| 2008 | Valencia Spain | Mbini Eq. Guinea | M 18 | Migrant | CSF | Second | Eﬂornithine | *T. b. gambiense* | 10 |
| 2009 | Sydney Australia | Adjumani Uganda | F 24 | Refugee | Brain biopsy | Second | Eﬂornithine | *T. b. gambiense* | 13 |
| 2009 | Castres France | Cocobeach Gabon | M 22 | Expat business | Blood smear /BM/CSF | Second | Eﬂornithine | *T. b. gambiense* | 10 |
| 2009 | Marseille France | Gabon | M NA | Expat business | Blood smear | First | Pentamidine | *T. b. gambiense* | 10 |
| 2009 | Netherlands | Angola | F 27 | Migrant | CSF | Second | Eﬂornithine | *T. b. gambiense* | 14 |
| 2009 | Braga Portugal | Catete Angola | M 55 | Expat business | Blood/CSF | Second | Eﬂornithine | *T. b. gambiense* | 10 |
| 2010 | Lisbon Portugal | Muxima Angola | F 2 | Migrant | Blood/CSF | Second | Eﬂornithine | *T. b. gambiense* | 10 |
| 2010 | Washington USA | Kumba Cameroon | M 37 | Migrant | Blood CCT | Second | Eﬂornithine | *T. b. gambiense* | 10 |
| 2010 | Cape Town South Africa | Bandundu DRC | M NA | Migrant | Blood CCT | Second | Eﬂornithine | *T. b. gambiense* | 10 |
| 2010 | Cape Town South Africa | Kikwit DRC | M 25 | Migrant | CSF | Second | Eﬂornithine | *T. b. gambiense* | 10 |
| 2001 | Johannesburg South Africa | Mayowasi Tanzania | M 40 | Tourist | Blood smear | Second | Pentamidine, Melarsoprol | *T. b. rhodesiense* | 15 |
| 2001 | Verona Italy | Serengeti Tanzania | M 33 | Tourist | Blood smear | First | Suramin | *T. b. rhodesiense* | 16 |
| 2001 | Bergamo Italy | Serengeti Tanzania | M 32 | Tourist | Blood smear | First | Pentamidine | *T. b. rhodesiense* | 16 |
| 2001 | Bradford UK | Serengeti Tanzania | F 44 | Tourist | Blood smear | First | Suramin | *T. b. rhodesiense* | 16 |
| 2001 | Stockholm Sweden | Serengeti Tanzania | M 41 | Tourist | Blood smear | First | Suramin | *T. b. rhodesiense* | 16 |
| 2001 | Johannesburg South Africa | Serengeti Tanzania | M 68 | Tourist | Blood smear | Second | Melarsoprol | *T. b. rhodesiense* | 16 |
| 2001 | Oslo Norway | Serengeti Tanzania | M 27 | Tourist | Blood smear | First | Suramin | *T. b. rhodesiense* | 16 |
| 2001 | Chester New York, USA | Serengeti Tanzania | M 56 | Tourist | Blood smear | Second | Melarsoprol | *T. b. rhodesiense* | 10 |
| 2001 | Florida USA | Serengeti Tanzania | M 71 | Tourist | Blood smear | First | Suramin | *T. b. rhodesiense* | 10 |
| 2001 | Amsterdam The Netherlands | Serengeti Tanzania | F 52 | Tourist | Blood smear | Second | Suramin, Melarsoprol | *T. b. rhodesiense* | 16 |
| 2001 | Johannesburg South Africa | Serengeti Tanzania | F 34 | Tourist | Blood smear | First | Suramin | *T. b. rhodesiense* | 15 |
| 2001 | Wyoming USA | Serengeti Tanzania | F 50 | Tourist | Blood smear | First | Pentamidine | *T. b. rhodesiense* | 10 |
| 2001 | Salem North Carolina, USA | Serengeti Tanzania | M 18 | Tourist | Blood smear | First | Suramin | *T. b. rhodesiense* | 10 |
| 2001 | Johannesburg South Africa | Serengeti Tanzania | M 29 | Tourist | Blood smear | First | Suramin | *T. b. rhodesiense* | 15 |
| 2001 | Leuven Belgium | Serengeti Tanzania | M 28 | Tourist | Blood smear | First | Suramin | *T. b. rhodesiense* | 17 |
| 2001 | Antwerp Belgium | Serengeti Tanzania | M 32 | Tourist | Blood smear | First | Suramin | *T. b. rhodesiense* | 10 |
| 2001 | USA | Serengeti, Tanzania | F 57 | Tourist | Blood smear | First | Suramin | *T. b. rhodesiense* | 10 |
| 2001 | Amsterdam The Netherlands | Tarangire Tanzania | M 57 | Tourist | Blood smear | First | Suramin | *T. b. rhodesiense* | 16 |
| 2001 | Amsterdam The Netherlands | Tarangire Tanzania | F 55 | Tourist | Blood smear | First | Suramin | *T. b. rhodesiense* | 16 |
| 2002 | London UK | L. Zambezi Zambia | M NA | Ranger | Blood smear | First | Suramin | *T. b. rhodesiense* | 10 |
| 2002 | Italy | Tanzania | M 33 | Tourist | Blood smear | First | Pentamidine, Suramin | *T. b. rhodesiense* | 16，17 |
| 2002 | Italy | Tanzania | M 30 | Tourist | Blood smear | First | Pentamidine | *T. b. rhodesiense* | 16，17 |
| 2002 | UK | Tanzania | F 44 | Tourist | NA | Second | Pentamidine | *T. b. rhodesiense* | 16 |
| 2002 | Switzerland | Tanzania | M 41 | Tourist | NA | First | Suramin | *T. b. rhodesiense* | 16 |
| 2002 | South Africa | Tanzania | M 68 | Tourist | NA | Second | Melarsoprol | *T. b. rhodesiense* | 16 |
| 2002 | Norway | Tanzania | F 27 | Researcher | NA | First | Suramin | *T. b. rhodesiense* | 16 |
| 2002 | Netherlands | Tanzania | M 60 | Tourist | Blood smear | First | Suramin | *T. b. rhodesiense* | 16 |
| 2002 | Netherlands | Tanzania | F 55 | Tourist | Blood smear | First | Suramin | *T. b. rhodesiense* | 18 |
| 2002 | Netherlands | Tanzania | F 53 | Tourist | Blood smear | Second | Suramin, Melarsoprol | *T. b. rhodesiense* | 10 |
| 2002 | Chennai India | Serengeti Tanzania | M 40 | Tourist | Blood smear | First | Suramin | *T. b. rhodesiense* | 19 |
| 2002 | Louisville Kentucky, USA | Serengeti Tanzania | M 40 | Tourist | Blood smear | Second | Melarsoprol | *T. b. rhodesiense* | 10 |
| 2003 | London UK | Serengeti Tanzania | M 9 | Tourist | Fluid chancre | First | Suramin | *T. b. rhodesiense* | 20 |
| 2003 | London UK | Serengeti Tanzania | M 14 | Tourist | Fluid chancre | First | Suramin | *T. b. rhodesiense* | 20 |
| 2003 | Netherlands | Tanzania | M 28 | Tourist | Blood smear | First | Suramin | *T. b. rhodesiense* | 17 |
| 2004 | Johannesburg South Africa | Kasungu Malawi | M 38 | Tourist | Blood ﬁlm | Second | Suramin, Melarsoprol | *T. b. rhodesiense* | 21 |
| 2004 | Johannesburg South Africa | Kasungu Malawi | M NA | Tourist | Blood smear | Second | Suramin, Melarsoprol | *T. b. rhodesiense* | 10 |
| 2004 | Bozeman Montana, USA | Mayowasi Tanzania | M 58 | Tourist | Blood smear | First | Pentamidine, Suramin | *T. b. rhodesiense* | 10 |
| 2004 | Pretoria South Africa | Serengeti Tanzania | F 52 | Tourist | Blood smear | Second | Suramin, Melarsoprol | *T. b. rhodesiense* | 22 |
| 2004 | Rochester Minnesota, USA | Serengeti Tanzania | F 61 | Tourist | Blood smear | Second | Pent/Suramin, Melarsoprol | *T. b. rhodesiense* | 23 |
| 2004 | UK | Tanzania | M 9 | Tourist | Lesion aspiration | First | Suramin | *T. b. rhodesiense* | 20 |
| 2004 | UK | Tanzania | M 14 | Tourist | Lesion aspiration and blood | First | Suramin | *T. b. rhodesiense* | 20 |
| 2005 | Johannesburg South Africa | Kariba Zimbabwe | M 60 | Ranger | Blood smear | First | Suramin | *T. b. rhodesiense* | 10 |
| 2005 | Johannesburg South Africa | Kasungu Malawi | M 26 | Soldier | Blood smear | First | Suramin | *T. b. rhodesiense* | 24 |
| 2005 | Johannesburg South Africa | Kasungu Malawi | M NA | Tourist | Blood smear | First | Suramin | *T. b. rhodesiense* | 10 |
| 2005 | Bethesda Maryland, USA | Serengeti Tanzania | F NA | Tourist | Blood smear | First | Suramin | *T. b. rhodesiense* | 10 |
| 2005 | Des Moines Iowa, USA | Serengeti Tanzania | M 71 | Tourist | Blood smear | Second | Suramin, Melarsoprol | *T. b. rhodesiense* | 10 |
| 2006 | Johannesburg South Africa | Q. Elizabeth Uganda | F 52 | Tourist | Blood smear | First | Suramin | *T. b. rhodesiense* | 10 |
| 2006 | Michigan, USA | Tarangire Tanzania | M 63 | Tourist | Blood smear | Second | Suramin, Melarsoprol | *T. b. rhodesiense* | 10 |
| 2006 | UK | Malawi | M 26 | Soldier | Blood smear | First | Suramin | *T. b. rhodesiense* | 25 |
| 2006 | Australia | Malawi | F 25 | Tourist | Blood smear | First | Suramin | *T. b. rhodesiense* | 26 |
| 2006 | Australia | Malawi | M 31 | Tourist | Blood smear | First | Suramin | *T. b. rhodesiense* | 26 |
| 2007 | Johannesburg South Africa | Kasungu Malawi | M NA | Tourist | Blood smear | First | Suramin | *T. b. rhodesiense* | 10 |
| 2007 | Johannesburg South Africa | Kasungu Malawi | M NA | Soldier | Blood smear | First | Suramin | *T. b. rhodesiense* | 10 |
| 2007 | Johannesburg South Africa | Kasungu Malawi | M NA | Ranger | Blood smear | First | Suramin | *T. b. rhodesiense* | 10 |
| 2007 | London UK | S. Luangwa Zambia | M NA | Tourist | Blood smear | First | Suramin | *T. b. rhodesiense* | 10 |
| 2007 | Johannesburg South Africa | Vwaza Malawi | F 25 | Tourist | Blood smear | First | Suramin | *T. b. rhodesiense* | 26 |
| 2007 | Johannesburg South Africa | Vwaza Malawi | M 31 | Tourist | Blood smear | First | Suramin | *T. b. rhodesiense* | 26 |
| 2008 | Lansing Michigan, USA | Mayowasi Tanzania | M 63 | Tourist | Blood smear | First | Suramin | *T. b. rhodesiense* | 10 |
| 2008 | Port Elizabeth South Africa | Serengeti Tanzania | M NA | Tourist | Blood smear | First | Suramin | *T. b. rhodesiense* | 10 |
| 2008 | London UK | Serengeti Tanzania | F 32 | Tourist | Blood smear | First | Pentamidine, Suramin | *T. b. rhodesiense* | 27 |
| 2008 | Amsterdam The Netherlands | Serengeti Tanzania | F 30 | Tourist | Blood smear | First | Suramin | *T. b. rhodesiense* | 28 |
| 2009 | Johannesburg South Africa | Mana Pools Zimbabwe | F 44 | Tourist | Blood smear | First | Suramin | *T. b. rhodesiense* | 10 |
| 2009 | Atlanta Georgia, USA | Mana Pools Zimbabwe | M 60 | Tourist | Blood smear | First | Suramin | *T. b. rhodesiense* | 10 |
| 2009 | Johannesburg South Africa | Nkhotakota Malawi | M 54 | Ranger | Blood smear | First | Suramin | *T. b. rhodesiense* | 10 |
| 2009 | Poznan Poland | Q. Elizabeth Uganda | M 61 | Tourist | Blood smear | First | Pentamidine | *T. b. rhodesiense* | 10 |
| 2009 | Du¨ sseldorf Germany | S. Luangwa Zambia | M 58 | Tourist | Blood smear | First | Suramin | *T. b. rhodesiense* | 10 |
| 2009 | Cedar Rapids Iowa, USA | Serengeti Tanzania | M NA | Tourist | Blood smear | First | Suramin | *T. b. rhodesiense* | 10 |
| 2009 | Johannesburg South Africa | Serengeti Tanzania | M 69 | Tourist | Blood smear | First | Suramin | *T. b. rhodesiense* | 10 |
| 2009 | Leiden The Netherlands | Serengeti Tanzania | F 25 | Tourist | Blood smear | First | Suramin | *T. b. rhodesiense* | 10 |
| 2009 | Tel Aviv Israel | Serengeti Tanzania | F 31 | Tourist | Blood smear | First | Suramin | *T. b. rhodesiense* | 29 |
| 2010 | Pretoria South Africa | Kasungu Malawi | M NA | Tourist | Blood smear | First | Suramin | *T. b. rhodesiense* | 10 |
| 2010 | London UK | Mana Pools Zimbabwe | F 55 | Tourist | Blood smear | Second | Suramin, Melarsoprol | *T. b. rhodesiense* | 10 |
| 2010 | Sao Paulo Brazil | Mana Pools Zimbabwe | M NA | Tourist | Blood smear | First | Pentamidine | *T. b. rhodesiense* | 10 |
| 2010 | Johannesburg South Africa | S. Luangwa Zambia | M 34 | Ranger | Blood smear | First | Suramin | *T. b. rhodesiense* | 10 |
| 2010 | Dallas Texas, USA | S. Luangwa Zambia | M NA | Tourist | Blood smear | First | Suramin | *T. b. rhodesiense* | 10 |
| 2010 | Liverpool UK | S. Luangwa Zambia | F NA | Tourist | Blood smear | First | Suramin | *T. b. rhodesiense* | 10 |
| 2010 | Johannesburg South Africa | S. Luangwa Zambia | M NA | Tourist | Blood smear | First | Suramin | *T. b. rhodesiense* | 10 |

NA: Information is unavailable on the case report.

**Table S4 Cases of human African trypanosomiasis (HAT) in non-endemic countries from 1990 to 2000.**

| **Year** | **Place of diagnosis** | **Place of infection** | **Sex/Age** | **Activity** | **Diagnosis** | **Stage** | **Treatment** | **Species** | **Reference** |
| --- | --- | --- | --- | --- | --- | --- | --- | --- | --- |
| 1991 | New Zealander | Nigeria, Gabon | M 32 | Ex-pat | Lymph, blood | First | Suramin, diﬂuoromethylornithine | *T. b. gambiense* | 30 |
| 1992 | French | Angola | M Young | Immigrant | NA | Second | Eﬂornithine | *T. b. gambiense* | 31 |
| 1994 | German | Nigeria | M 54 | Tourist | CSF/ blood smear | Second | suramin, prednisolone, melarsoprol | *T. b. gambiense* | 32 |
| 1995 | Dutch | Cameroon | F 52 | Immigrant | CSF | Second | Suramin, melarsoprol | *T. b. gambiense* | 33 |
| 1996 | Italian | Zaire | M 32 | NA | Blood | First | Eﬂornithine | *T. b. gambiense* | 28 |
| 1999 | French | Gabon | M 45 | Ex-pat | Blood | First | Pentamidine | *T. b. gambiense* | 34 |
| 1990 | Swiss | Rwanda | M NA | tourist | Blood, CSF | Second | Melarsoprol | *T. b. rhodesiense* | 35,36 |
| 1990 | Swiss | Rwanda | M NA | tourist | Blood | First | Suramin | *T. b. rhodesiense* | 35,36 |
| 1991 | American | Tanzania, Kenya, Rwanda | M 49 | tourist | Blood | First | Pentamidine, suramin | *T. b. rhodesiense* | 37 |
| 1994 | French | Rwanda | M NA | solider | CSF | Second | Melarsoprol | *T. b. rhodesiense* | 38 |
| 1994 | French | Rwanda | M NA | solider | Blood, medulla | Second | Melarsoprol | *T. b. rhodesiense* | 38 |
| 1996 | Mexico | Kenya | M 57 | tourist | Blood, lesion exudate, CSF | Second | Pentamidine, melarsoprol | *T. b. rhodesiense* | 39 |
| 1997 | French | Rwanda | M 30 | tourist | Blood, marrow, CSF | Second | Melarsoprol | *T. b. rhodesiense* | 40 |
| 1999 | American | Tanzania | M 41 | tourist | Blood, CSF | Second | Suramin, melarsoprol | *T. b. rhodesiense* | 41 |
| 1999 | American | Tanzania | F 54 | tourist | Blood | First | Suramin | *T. b. rhodesiense* | 42 |
| 1999 | American | Tanzania | M 49 | tourist | Blood | First | Suramin | *T. b. rhodesiense* | 42 |
| 2000 | Johannesburg South Africa | Kasungu Malawi | M 45 | Tourist | Blood smear | First | Suramin | *T. b. rhodesiense* | 3 |
| 2000 | Massachusetts USA | Serengeti Tanzania | M 37 | Tourist | Blood smear | First | Suramin | *T. b. rhodesiense* | 43 |
| 2000 | London UK | S. Luangwa Zambia | M 51 | Tourist | Blood smear | First | Suramin | *T. b. rhodesiense* | 44,45 |
| 2000 | London UK | Serengeti Tanzania | M 30 | Tourist | Blood smear | First | Suramin | *T. b. rhodesiense* | 44,45 |
| 2000 | Salem Ohio, USA | Mayowasi Tanzania | M 47 | Tourist | Blood smear | First | Suramin | *T. b. rhodesiense* | 3 |

**REFERENCES**

1. Sahlas DJ, MacLean JD, Janevski J, Detsky AS (2002) Clinical problem-solving out of Africa. *N Engl J Med* 347, 749-753.

2. Rocha G, Martins A, Gama G, Brandão F, Atouguia J (2004) Possible cases of sexual and congenital transmission of sleeping sickness. *Lancet* 363, 247.

3. Migchelsen SJ, Büscher P, Hoepelman AI, Schallig HD, Adams ER (2011) Human African trypanosomiasis: a review of non-endecmic cases in the past 20 years*. Int J Infect Dis* 15, e517-524.

4. Landron C, Roblot F, Le Moal G, Becq-Giraudon B. (2003) African trypanosomiasis acquired in an urban area. *Eur J Intern Med* 14, 390-391.

5. Ehrhardt S, Lippert U, Burchard GD, Sudeck H. (2006) Orchitis as an unusual manifestation of human African trypanosomiasis. *J Infect* 52, e31-e33.

6. Hope-Rapp E, Moussa Coulibaly O, Klement E, Danis M, Bricaire F, Caumes E (2009) Chancres cutanés relevant une trypanosomose africaineá *Trypanosoma brucei gambiense* [Double trypanosomal chancre revealing West African trypanosomiasis in a Frenchman living in Gabon]. *Ann Dermatol Venereol* 136, 341-345.

7. Bisoffi Z, Beltrame A, Monteiro G, Arzese A, Marocco S, Rorato G, Aneselmi M, Viale P (2005) African trypanosomiasis Gambiense, Italy. *Emerg Infect Dis* 11, 1745-1747.

8. Kager PA, Schipper HG, Stam J, Majoie CB (2009) Magnetic resonance imaging findings in human African trypanosomiasis: a four-year follow-up study in a patient and review of literature. *Am J Trop Med Hyg* 80, 947-952.

9. Gautret P, Clerinx J, Caumes E, Simon F, Jesenius M, Loutan L, Schlagenhauf P, Castelli F, Freedman D, Miller A, Bronner U, Parola P (2009) Imported human African trypanosomiasis in Europe, 2005-2009. *Euro Surveill* 14, 19327.

10. Simarro PP, Franco JR, Cecchi G, Paone M, Diarra A, Postigo JAR, Jannin JG (2012) Human African trypanosomiasis in non-endemic countries (2000-2010). *J Travel Me*d 19, 44-53.

11. Ezzedine K, Darie H, Le Bras M, Malvy D. (2007) Skin features accompanying imported human African trypanosomiasis: hemolymphatic *Trypanosoma gambiense* infection among two French expatriates with dermatologic manifestations. *J Travel Med* 14, 192-196.

12. Cherian P, Junckerstorff RK, Rosen D, Kumarasinghe P, Morling A, Tuch P, Raven S, Murray RJ, Heath CH (2010) Late stage human African trypanosomiasis in a Sudanese refugee. *Med J Aust* 192, 417-419.

13. Liu AP, Chou S, Gomes L, Ng T, Salisbury EL, Walker GL, Packham DR (2010) Progressive meningoencephalitis in a Sudanese immigrant. *Med J Aust* 192, 413-416.

14. Kager PA, Schipper HG, Stam J, Majoie CB (2009) Magnetic resonance imaging findings in human African trypanosomiasis: a four-year follow-up study in a patient and review of the literature. *Am J Trop Med Hyg* 80, 947-952.

15. Dini LA, Blumberg LH, Frean JA (2001) East African trypanosomiasis: a re-emerging threat to South African tourists (poster). Paper presented at: joint congress of the infectious diseases and sexually transmitted diseases societies of southern Africa, stellenbosch, south Africa, December 2001.

16. Jelinek T, Bisoffi Z, Bonazzi L, Thiel Pv, Bronner U, Frey Ad, Gundersen SG, McWhinney P, Ripamonti D (2002) Cluster of African trypanosomiasis in travelers to Tanzanian national parks. *Emerg Infect Dis* 8, 634-635.

17. Callens S, Van Wijngaerden E, Clerinx J, Colebunders B (2003) Three patients with African sleepingsickness following a visit to Tanzania. *Ned Tijdschr Geneeskd* 147: 581.

18. Mendonca Melo M, Rasica M, van Thiel PP, Richter C, Wismans PJ (2002) Three patients with African sleeping sickness following a visit to Tanzania. *Ned Tijdschr Geneeskd* 146, 2552-2556.

19. Gopalakrishnan R, Easow JM (2003) East African sleepingsickness in Chennai. *J Assoc Physicians India* 51, 302-303.

20. Faust SN, Woodrow CJ, Patel S, Snape M, Chiodini PL, Tudor-Williams G, Lyall EGH (2004) Sleeping sicknessin brothers in London. *Pediatr Infect Dis J* 23, 879-881.

21. Checkley AM, Pepin J, Gibson WC, Taylor MN, Jäger HR, Mabey DC (2007) Human African trypanosomiasis: diagnosis, relapse and survival after severe melarsoprol-induced encephalopathy. *Trans R SocTrop Med Hyg* 101, 523-526.

22. Braakman HM, van de Molengraft FJ, Hubert WW, Boerman DH (2006) Lethal African trypanosomiasis in a traveler: MRI and neuropathology. *Neurology* 66, 1094-1096.

23. Kumar N, Orenstein R, Uslan DZ, Berbari EF, Klein CJ, Windebank AJ (2006) Melarsoprol associated multifocal inflammatory CNS illness in African trypanosomiasis. *Neurology* 66, 1120-1121.

24. Croft AM, Kitson MM, Jackson CJ, Minton EJ, Friend HM (2007) African trypanosomiasis in a British soldier. *Mil Med* 172, 765-769.

25. Croft AM, Jackson CJ, Friend HM, Minton EJ (2006) African trypanosomiasis in a British soldier. *J R Army Med Corps* 2006 152, 156-160.

26. Darby JD, Huber MG, Sieling WL, Spelman DW (2008) African trypanosomiasis in two short-term Australian travelers to Malawi. *J Travel* 15, 375-377.

27. Nadjm B, Van Tulleken C, Macdonald D, Chiodini PL (2010) East African trypanosomiasis in a pregnant traveller. *Emerg Infect Dis* 2009; 15: 1866-1867.

28. Claessen FA, Blaauw GJ, van der Vorst MJ, et al. Tryps after adventurous trips. *Neth J Med* 68, 144-145.

29. Meltzer E, Leshem E, Gutman D, Schwartz E (2009) Human African trypanosomiasis in an Israeli traveller. Paper presented at: annual meeting 2009 & workshop on vectors and vector-borne diseases. Israel Society for Parasitology, Protozoology and Tropical Diseases, Kfar Hamaccabiah, Ramat Gan, December 2009, 27-28.

30. Scott JA, Davidson RN, Moody AH, Bryceson AD (1991) Diagnosing multiple parasitic infections: trypanosomiasis, loiasis and schistosomiasis in a single case. *Scand J Infect Dis* 23, 777-780.

31. Blanchot I, Dabadie A, Tell G, Guiguen C, Faugère B, Plat-Pelle AM, Roussey M (1992) Recurrent fever episodes in an African child: diagnostic difficulties of trypanosomiasis in France. *Pediatrie* 47, 179-183.

32. Damian MS, Dorndorf W, Burkardt H, Singer I, Leinweber B, Schachenmayr W (1994) Polyneuritis and myositis in *Trypanosoma gambiense* infection. *Dtsch Med Wochenschr* 119, 1690-1693.

33. Otte JA, Nouwen JL, Wismans PJ, Beukers R, Vroon HJ, Stuiver PC (1995) African sleeping sickness in the Netherlands. *Ned Tijdschr Geneeskd* 139, 2100-2104.

34. Iborra C, Danis M, Bricaire F, Caumes E (1999) A traveler returning from central Africa with fever and a skin lesion. *Clin Infect Dis* 28, 679-680.

35. Gautret P, Clerinx J, Caumes E, Simon F, Parola P (2009) Imported human African trypanosomiasis in Europe, 2005-2009. *Euro Surveill* 14, 19327.

36. Braendli B, Dankwa E, Junghanss T (1990) East African sleeping sickness (*Trypanosoma rhodesiense* infection) in 2 Swiss travelers to the tropics. *Schweiz Med Wochenschr* 120, 1348-1352.

37. Panosian CB, Cohen L, Bruckner D, Berlin G, Hardy WD (1991) Fever leukopenia, and a cutaneous lesion in a man who had recently traveled in Africa. *Rev Infect Dis* 13, 1131-1138.

38. Montmayeur A, Brosset C, Imbert P, Buguet A (1994) The sleep–wake cycle during *Trypanosoma brucei rhodesiense* human African trypanosomiasis in 2 French parachutists. *Bull Soc Pathol Exot* 87, 368-371.

39. Ponce-de-León S, Lisker-Melman M, Kato-Maeda M, Gamboa-Domínguez A, Ontiveros C, Behrens RH, González-Ruiz A (1996) *Trypanosoma brucei rhodesiense* infection imported to Mexico from a tourist resort in Kenya. *Clin Infect Dis* 23, 847-848.

40. Sabbah P, Brosset C, Imbert P, Bonardel G, Jeandel P, Briant JF (1997) Human African trypanosomiasis: MRI. *Neuroradiology* 1997; 39: 708-710.

41. Malesker MA, Boken D, Ruma TA, Vuchetich PJ, Murphy PJ, Smith PW (1999) Rhodesian trypanosomiasis in a splenectomized patient*. Am J Trop Med Hyg* 61, 428-430.

42. Sinha A, Grace C, Alston WK, Westenfeld F, Maguire JH (1999) African trypanosomiasis in two travelers from the United States. *Clin Infect Dis* 29, 840-844.

43. Moore AC, Ryan ET, Waldron MA (2002) A 37-year-old man with fever, hepatosplenomegaly, and a cutaneous foot lesion after a trip to Africa–East African trypanosomiasis (*Trypanosoma brucei rhodesiense* infection). *N Eng J Med* 346, 2069-2076.

44. Moore DAJ, Edwards M, Escombe R, Agranoff D, Bailey JW, Squire B, Chiodini PL (2002) African trypanosomiasis in travelers returning to the United Kingdom. *Emerg Infect Dis* 8, 74-76.

45. Jones J (2000) African sleeping sickness returns to UK after four years. *BMJ*  321, 1177.
